# Accuracy of Direct Composite Veneers via Injectable Resin Composite and Silicone Matrices in Comparison to Diagnostic Wax-Up

**DOI:** 10.3390/jfb14010032

**Published:** 2023-01-05

**Authors:** Vasiliki Kouri, Domna Moldovani, Efstratios Papazoglou

**Affiliations:** 1 Operative Dentistry, National and Kapodistrian University, 11527 Athens, Greece; 2 Private Practice, 14341 Athens, Greece; 3 School of Dentistry, National and Kapodistrian University, 11527 Athens, Greece

**Keywords:** resin composite veneers, injection technique, index technique

## Abstract

Purpose: To evaluate the discrepancy between the diagnostic wax-up and the resulting direct veneers using different matrices. Materials and method: A total of 48 identical misaligned models, 12 wax-up models and one ‘every other tooth’ wax-up model were 3D printed. Group 1: Transparent silicone matrices with holes for the injection of the flowable composite. Group 2: The same procedure as group 1, but the first three teeth were restored using the matrix constructed from the ‘every other tooth’ wax-up model. Group 3: Transparent silicone matrices cut for each tooth and preheated resin composite. Group 4: The same procedure as group 3, but the loaded matrix was placed first in the clear plastic tray, which was used for the matrix’s fabrication. Group 5: Wax-up models (control group). Scans from the veneers were superimposed with the scans from the wax-up and compared using the Patient Monitoring software. Measurements were made at the incisal, middle and cervical third. Kruskal-Wallis test and Dunn post-hoc test (*p* < 0.05) were used to analyze the results. Results: No statistically significant differences were found between groups 1 and 2 and the control. Group 3 was different from the control in the incisal and middle third, and group 4 was different in the cervical third. Conclusions: (1) Use of one or two matrices and the injection of flowable composite were accurate techniques. (2) Use of a matrix for each tooth combined with a pre-heated composite was the most inaccurate technique, but the use of the clear tray in combination with the matrix improved the accuracy.

## 1. Introduction

The aim of restorative dentistry is the biological, functional and esthetic oral rehabilitation due to caries, periodontal disease, trauma, shape and color disharmonies or tooth wear. Focusing on the anterior teeth, depending on the remaining tooth substrate and the changes that need to be made, direct resin composite restorations play an important role. Resin composite can restore the part of the tooth that is missing due to caries or trauma. Furthermore, resin composite can be used to change their shape or to close diastemas to improve esthetics [1,2]. By restoring the anterior teeth with veneers, it is possible to make more changes in the shape, the dimensions, the inclination and the color of the teeth. Veneers usually are the preferable option to improve esthetics, except for cases in need of crowns because of the decreased tooth structure [3,4]. 

Veneers can be made of ceramic or resin composite. Veneers made of resin composite can be constructed by using two different techniques: direct or indirect technique. Resin composite restorations undergo more changes in esthetic properties than ceramics [5,6,7,8,9,10,11,12]. Nevertheless, direct resin composite veneers, usually, require less preparation and, in several cases, there is no need for tooth preparation. Therefore, these restorations increase the longevity of the teeth [13].

Clinician decisions about which technique is best to apply depend on the case and his or her own treatment philosophy and skills. From a clinician’s perspective, treatment with direct resin composite veneers is completed in one or two appointments. The clinician needs specific training and skills to be able to create highly esthetic direct restorations by customizing the shapes, the morphology, the color and the opacities [1,2]. On the other hand, the creation of the ceramic veneers is carried out by the dental technician, which results in an increase in treatment costs, and many patients may not be able to afford it. Focusing on esthetic longevity, ceramic veneers have excellent shade and gloss stability, while direct resin composite veneers need repolishing once per year on average. With the direct technique, there is more often no need for tooth preparation than with the indirect technique, where rarely ‘no prep’ veneers are carried out [13].

It is a fact that the free-hand direct technique requires a highly skilled clinician, and the final result is not predictable. Several clinicians have recommended restorative concepts to overcome this difficulty, so the workflow becomes easier, stress free and predictable. One popular approach for anterior extended restorations recommended starting the restorative procedure with the construction of a silicone palatal matrix based on a diagnostic wax-up. The silicone matrix is extended to adjacent unrestored teeth to ensure precise fit and positioning. The process is started by applying the palatal increment of resin composite into the silicone matrix, resulting in the palatal surface of the restoration. Then, the clinician restores the proximal surfaces with sectional matrices and the buccal surfaces with a free-hand technique [14].

**Figure 1:**
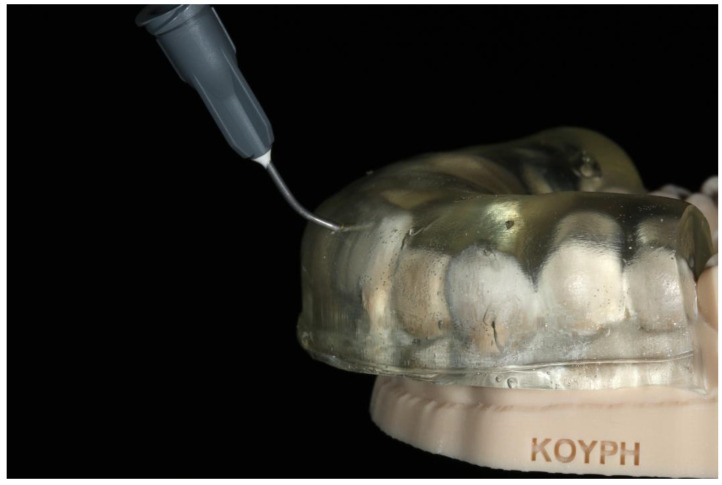
A clear polyvinyl siloxane matrix with openings incisally, seated on the 3D printed diagnostic wax-up cast.

Additional published concepts have been based on the idea of constructing direct composite veneers via matrices. In two articles, Terry et al. described the resin composite injection technique, which mainly focuses on the rehabilitation of the anterior teeth. The restorative concept included the construction of a clear polyvinyl siloxane matrix by replicating the diagnostic wax-up using a nonperforated tray. After the curing of the matrix material, small openings are made in the middle of the incisal edge for the injection of the flowable resin composite (Figure 1). Before the application of the adhesive protocol to one tooth buccal and lingual surfaces, the adjacent teeth are separated using teflon tape. After the adhesive procedure, the matrix is placed over the anterior teeth and the flowable resin composite is injected through the opening above the tooth to be restored. Light curing takes place and, after the removal of the matrix, the last step is the finishing and polishing of the restoration. The same procedure is repeated for the restorations of the adjacent teeth. This treatment concept has been suggested to restore anterior fractured teeth, class III and IV cavities, composite veneers and pediatric composite crowns. Furthermore, the injection technique is useful for space-closing after orthodontic treatment, the fabrication of tooth- and implant-supported provisionals, and the restoration of fractured or missing denture teeth. In addition, the workflow can be used to restore worn occlusal surfaces of the posterior teeth [15,16]. This restorative concept, which is described above, has quickly become very popular among operative dentists all over the world. Some have published cases treated using this technique [17,18,19,20], and others applied the technique and suggested some modifications.

Coachman et al. suggested an improved direct injection technique with flowable resin composites. The procedure was the same as described above, with the difference that two wax-up casts and two indexes were fabricated for the restorations. The first cast was obtained by making a wax-up of every other tooth, and then the first index was obtained by taking an impression with clear polyvinyl siloxane. The second cast was obtained by making a full wax-up of the teeth to be restored, and the second index by taking an impression from that cast. It is very important to note that this procedure can only be precise with a digital workflow, because it is very difficult for the technician to make two identical wax-up casts using the analog waxing technique. The goal of this improved technique was to block flowable resin composite from flowing onto the adjacent teeth, and to create a correct mesial and distal surface because of the tight seal between the waxed and unwaxed teeth of the first index. This way, the finishing and polishing time was decreased, and the treatment procedure was easier for the clinician [21].

Similarly, Gestakovski published a clinical case restored by using the injection technique with a two-year follow-up. The workflow had a modification compared to the original technique, which was the separation of the mock-up for the creation of space holders. With this modification, the composite prevented flow onto adjacent teeth [22]. Similarly, Kole et al. published an injection technique case and recommended a modification regarding the construction of the clear matrix. The first step was the construction of an index made of putty impression material and the creation of a window by cutting the index above the teeth to be restored. Then, clear polyvinyl siloxane was injected into the window and a cellophane sheet was adapted to the material to shape the index [23].

**Figure 2:**
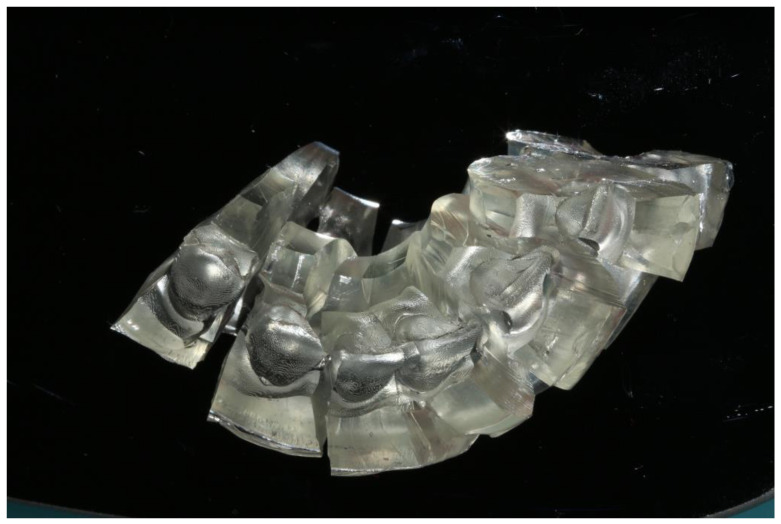
The index separately cut for each tooth.

**Figure 3:**
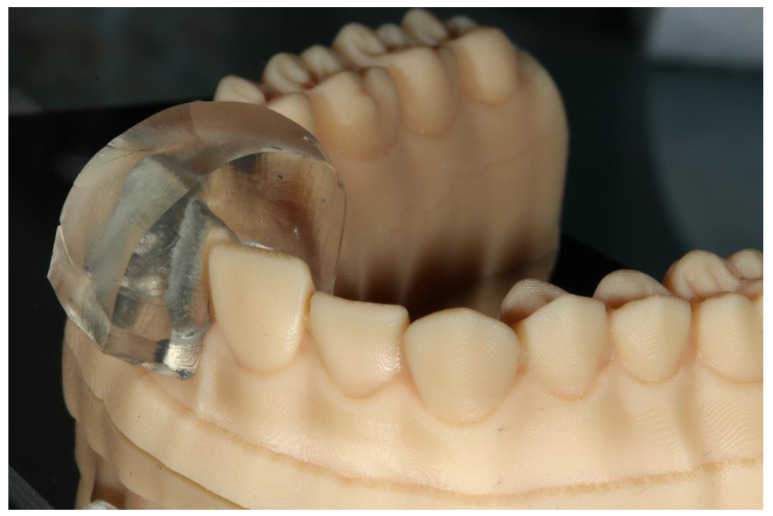
The fitting of the index for each tooth on the cast.

Ammannato et al. suggested ‘the index technique’, which was implemented in full mouth rehabilitation with conventional pre-heated resin composites. This technique was recommended for restoring worn teeth in an additive way, without preparation of the teeth. The treatment procedure began by restoring the upper and lower anterior teeth. The first step was the creation of the indexes by injecting a transparent silicone over the diagnostic wax-up. After polymerization, the index had to be separately cut for each tooth (Figure 2 and Figure 3). Optionally, a hole was created with a small round bur at each index to allow the free flow of the material. Following this, the adjacent teeth were protected with steel matrix bands and the adhesive protocol followed. Then, the preheated resin composite was placed on the tooth and the index was placed on the tooth for the precise fit by palatally and buccally pressuring the individual index. The excess material was removed with a probe and light curing took place through the transparent matrix. After the removal of the matrix, the next step was finishing and polishing, and the same procedure took place for the adjacent teeth of the sextant and the anterior lower teeth. The next step was to restore the posterior teeth at each sextant following the workflow described above. The patient was recalled for checking the occlusion again a few days later [24].

The use of matrices, resulting from the wax-up cast, aimed to keep the construction of direct resin composite veneers a simple and easy procedure for everyday dental practice. So far, there has been no research, in vitro or in vivo, to compare the different techniques described above regarding the accuracy of transferring the wax-up to the final restorations.

The purpose of this study was to evaluate the discrepancy between the diagnostic wax-up and the resulting direct veneers with resin composite via different matrices. The null hypothesis of the present study was that there is no dimensional difference between the resulting restorations with the different techniques and the diagnostic wax-up.

## 2. Materials and Methods

### 2.1. Casts Fabrication

**Figure 4:**
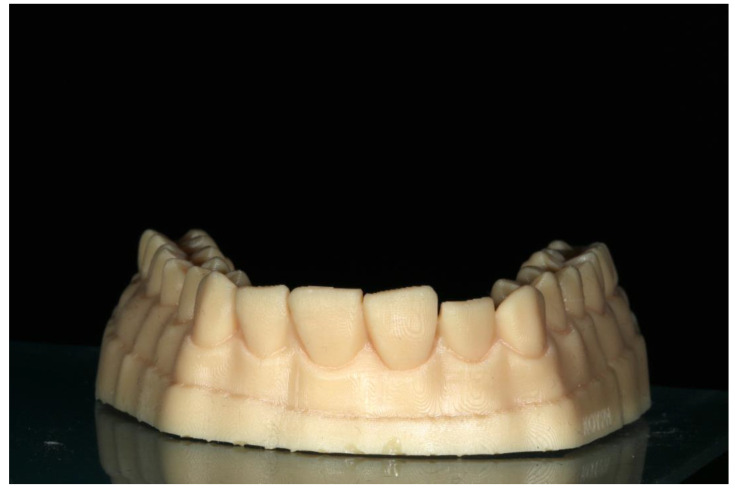
The first 3D-printed cast with six misaligned anterior teeth, which was used as the clinical case to be restored.

For the purpose of the experiment, a unique plastic model with six misaligned anterior teeth (Dentalstore, Milano, Italy) was used to simulate a clinical case to be restored. The model was scanned with a laboratory scanner (3Shape E3, Copenhagen, Denmark). According to the manufacturer, the accuracy level was approximately ±7 μm. Then, the first cast was printed using a 3D printer (Asiga Max UV, Sydney, Australia) (Figure 4) with 62 μm pixel resolution and 100 μm layer thickness, according to the manufacturer. The casting material was the 3D printer resin Optiprint Model, in golden brown color (Dentona, Dortmund, Germany).

**Figure 5:**
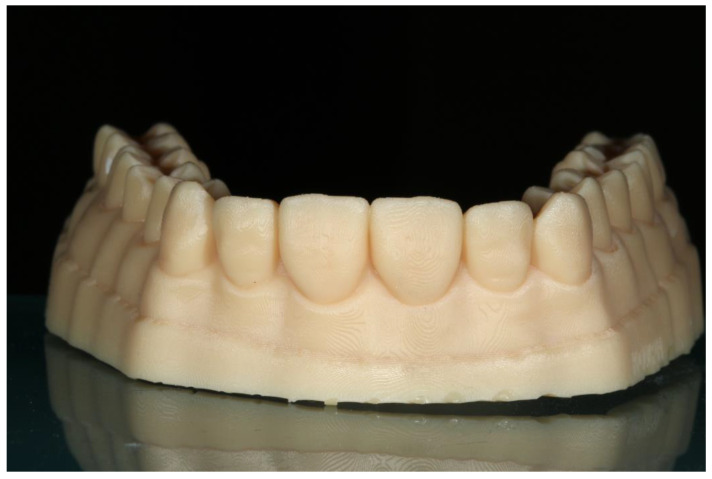
The complete wax-up 3D-printed cast.

**Figure 6:**
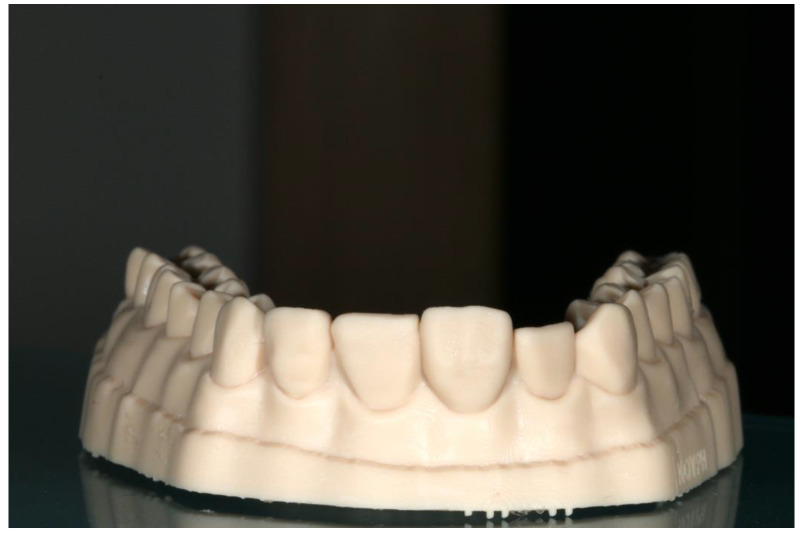
The wax-up 3D-printed cast after the deactivation of every other tooth from the complete wax-up.

G*Power 3.1.2 (Faul F., Erdfelder E., Lang A.G., Buchner A., Germany, Heinrich Heine University Dusseldorf, Germany) was used to compute the sample size needed for each group. For the sample size computation, the parameters used were: probability of type I error (two-tailed) 0.05; power 0.8; and effect size 0.5. The results indicated a minimum of 11 specimens in each group. Therefore, 12 specimens were fabricated for each of the 5 groups. The first printed cast was scanned and 48 identical specimens were 3D printed. Then, an additive digital wax-up was created on the 6 misaligned anterior teeth of the initial cast using EXOCAD. The next step was the 3D printing of 12 wax-up casts which constituted the control group and were used for the construction of the matrices (Figure 5). A second 3D-printed wax-up cast was produced by using the same software after the deactivation of every other tooth from the complete wax-up (Figure 6).

Before the restorative procedure, all specimens of the four researched groups and the control group were scanned, and the STL files were compared to the STL file of the initial printed cast, which was also scanned. This process was used for the measurement of the precision (repeatability) of the final measurements. Patient monitoring software (TRIOS Patient Monitoring 21.2 for Dental Desktop 1.7.27 3Shape, Copenhagen, Denmark) was used to compare STL files via superimposition. After the procedure, the inter-class correlation coefficient was estimated at 0.998, and the intra-class correlation coefficient was estimated at 0.882. The 3D-printed casts were stored in a dark room with stable temperature and humidity conditions. The temperature was adjusted to 20 °C and the humidity was 50%.

### 2.2. Matrices Construction

**Figure 7:**
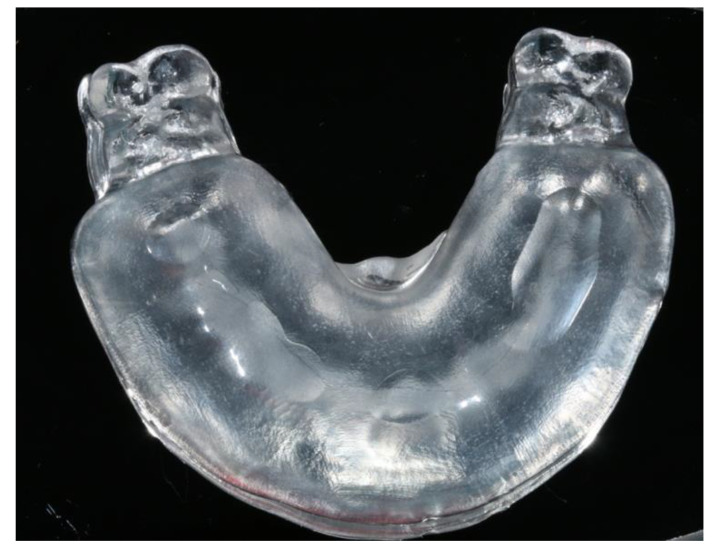
The 3D-printed clear tray which was designed to ensure uniform thickness of the matrices at 6 mm.

The 3D-printed casts that made up the control group were used for index construction. In an attempt to eliminate the effect of the matrix’s thickness on the final restorations, a clear tray designed in EXOCAD was 3D printed (Figure 7). The tray was designed to ensure a uniform matrix thickness of 6 mm. A study regarding the effect of silicone thickness on the Shore hardness of elastomers showed that the thickness should be at least 6 mm [25].

The tray had stops bilaterally on the first and second molars and one stop on the anterior palatal mucosa. The resulting matrices had a uniform thickness of 6 mm and extended to the second premolar at each side. All matrices were made of clear polyvinyl siloxane 60 Shore (Exaclear, GC Corporation, Tokyo, Japan). In total, 48 identical clear silicone matrices were constructed based on the complete wax-up models and 12 matrices based on the ‘every other tooth’ wax-up model. All matrices were constructed on the same day and were used for the restorations after 24 h for more complete polymerization reaction. The air temperature was 23 °C and the setting time on the model was 10 min.

### 2.3. Researched Groups

The restorative procedure for each technique was carried out as described below:

- Group 1: The direct veneers were made using the flowable resin composite G-Aenial universal injectable (GC Corporation, Tokyo, Japan) in A2 color. The matrices which were constructed on the complete wax-up models were used. Small openings were made onto the matrices using a dispensing tip in the middle of the buccal surface, too close to the incisal edge, for the injection of the flowable composite. After the fitting of the matrix, the flowable resin composite was injected into the opening for every other tooth until the material filled the space for each veneer. Polymerization was performed (Bluephase Style light curing unit, Ivoclar, Vivadent) for 60 s on each tooth at an intensity of 1100 mW/cm^2^. Then, the matrix was removed to simulate the clinical procedure and additional polymerization was performed for 40 s on each tooth. The matrix was seated again to restore the remaining three teeth.
- Group 2: The restorative procedure was as described above, but there was one modification. The first three teeth were restored using the matrix constructed from the ‘every other tooth’ wax-up model, and the remaining three teeth were restored by injecting composite through the matrix constructed from the complete wax-up model.
- Group 3: In this group, the matrices constructed on the complete wax-up model were used. The matrices were cut at the interproximal of each tooth to achieve a matrix for every tooth, and a small opening was created with the dispensing tip to allow the material to flow through. The veneers were constructed using the conventional hybrid resin composite Gradia Direct in A2 color (GC Corporation, Tokyo, Japan). The composite heating conditioner Ena Heat (MICERIUM, Italy) (voltage: 12 V, current: 1 A, power: 12 W) was used to preheat the material. The selected heating temperature was 55 °C and the weight of each resin composite dose was 0.15 g, which was estimated via a pilot test to be enough for each veneer. The index loaded with composite was placed on the respective tooth by palatal and buccal pressing. Before light curing, the excesses were removed and, after the polymerization process, the same procedure was performed to restore the rest of the teeth.
- Group 4: The restorative procedure was as described above, with an alteration. First, the silicone matrix was placed in the clear tray, which was used for the matrix fabrication, and then, that combination loaded with composite resin, was placed on each tooth.
- Group 5: The control group consisted of the printed wax-up models.

The construction of the matrices, the execution of all restorative procedures and all measurements were accomplished by the same investigator (V.K.).

### 2.4. Quantitative Analysis

**Figure 8:**
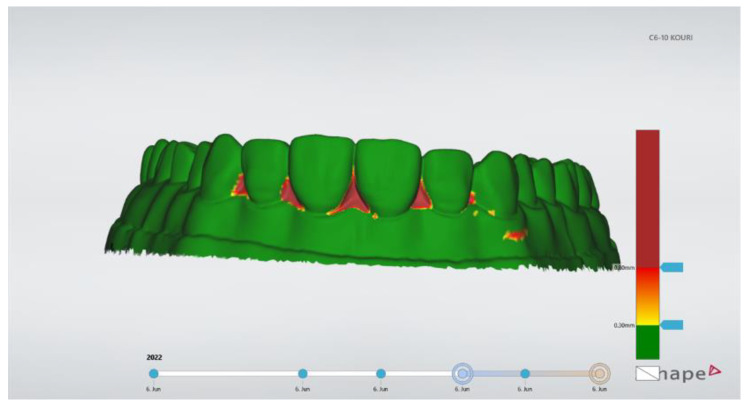
The appearance of 3D color-coded map verified the alignment of the imported models.

**Figure 9:**
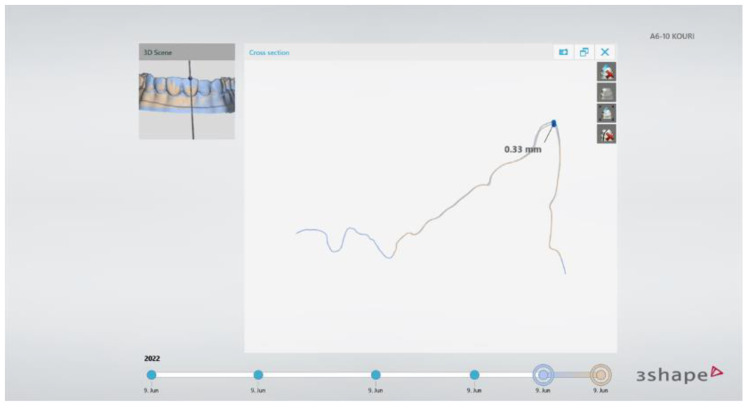
Representative cross-section plane in the middle of each tooth and appearance of the window with the tooth’s profile where the linear measurements took place.

After the completion of the direct veneers of the four research groups, the specimens were scanned using the lab scanner and STL files were created. Those STL files were imported to the patient monitoring software (TRIOS Patient Monitoring) for data analysis. The superimposition process was used based on the unrestored posterior teeth’s surfaces using three-point alignment. Best fit alignment was not used since it produced significantly higher alignment errors compared to the three-point alignment [26]. The three points selected were on teeth #14, #17, and #26. By choosing the color-coded 3D map, it was possible to verify the precise alignment of the two models. The posterior teeth had to be totally green, which meant differences between the models smaller than 0.30 mm (Figure 8). Then, the width of the tooth was measured and a cross-section plane was vertically set to the labial surface and at the buccal-lingual direction, exactly in the middle of each anterior tooth (#13–23). At the tooth profile cross-section window, the contour tracings of the teeth were automatically carried out by the software and linear measurements of the aligned models were taken (Figure 9). This procedure has been previously published by Moldovani et al. [27]. Three measurements for each tooth were made at the labial surface: incisal, middle and cervical. Finally, 1080 measurements were made. Three on every anterior tooth (#13–23) for twelve models or each of the five groups. According to the manufacturer, the measurement uncertainty was 50 μm when the scans were materialized by using the intra oral scanner.

### 2.5. Statistical Analysis

The Shapiro-Wilk test was run to examine if the data were normally distributed (*p* = 0.798). As the data did not follow a normal distribution, the non-parametric Kruskal-Wallis test and the Dunn’s post-hoc test were executed to compare the five groups for the incisal, middle and cervical thirds. The alpha level was set to 0.05 and significance values were adjusted by the Bonferroni correction for multiple tests. SPSS Statistics 26.0 (IBM Corp., Armonk, NY, USA) was used to carry out the statistical analysis.

## 3. Results

**Figure 10:**
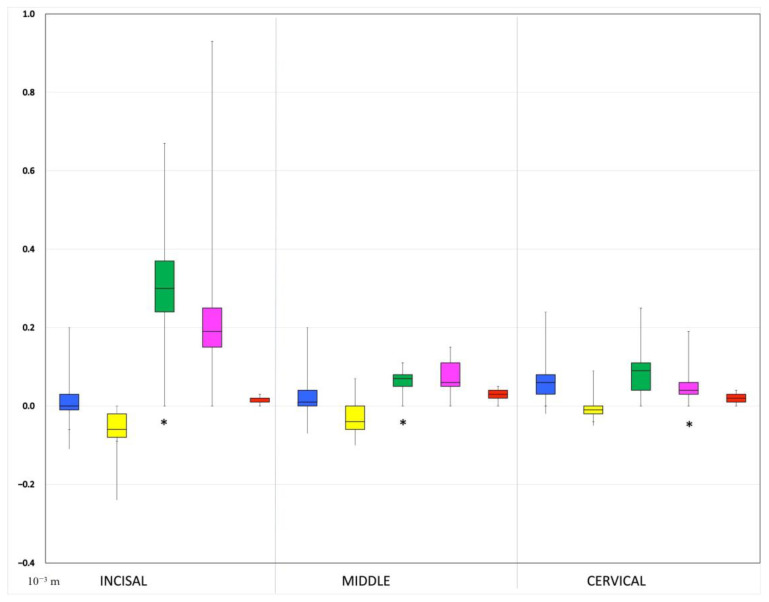
Box-plots showing the linear dimensional accuracy between the 5 groups at the incisal, middle and cervical third. Statistically significant differences compared to control group are marked with *. The whiskers show the range of the obtained values, the outliers, the interquartile range (colorful box) and the median (horizontal black line). The groups have different colors: **Blue**: Group 1, **Yellow**: Group 2, **Green**: Group 3, **Pink**: Group 4, **Red**: Group 5 (control).

No statistically significant differences were found between groups 1 and 2 and the control group (group 5) in all labial surface thirds. Regarding the comparison of group 3 and the control group, a statistically significant difference was identified in the incisal third and in the middle third, while the discrepancy in the cervical third was not statistically significant. The comparison of group 4 and the control group pointed out exactly the opposite results compared to the comparison of group 3 and the control group. Specifically, a statistically significant discrepancy was identified only in the cervical third of the labial surface. The largest discrepancy was observed in group 3 at the incisal third (median: 450 μm, IQR: 70 μm), followed by the middle third of the same group (median: 200 μm, IQR: 20 μm) and the cervical third of group 4 (median: 120 μm, IQR: 0 μm) (Figure 10).

## 4. Discussion

The results of the present study showed that the original injectable resin technique and its modification are two similar restorative techniques which accurately lead from the diagnostic wax-up to the direct resin composite veneers. These techniques were described in group 1 and group 2, and included transparent silicone matrices and the injection of a flowable resin composite. The discrepancies at the labial surfaces were not statistically significant between each of the two groups and the control group, as well as between the two. This finding demonstrates that the clinician is able to construct direct veneers almost identical to the diagnostic wax-up using the injection technique. This finding is very important for daily clinical practice, since the free-hand technique requires a highly skilled dentist and increased chairside time.

The difference between the two techniques using a transparent matrix was the use of a second transparent silicone matrix in group 2. The two matrices had three more anterior teeth as vertical stops to verify their precise fitting. However, the results demonstrated that this technique modification was not crucial enough to result in any significant difference. An advantage of the technique that uses two different matrices is the creation of more detailed distal and mesial surfaces, but that point was not evaluated in this study.

The most inaccurate group was group 3 at the incisal third, followed by the middle third, which were bulkier than in the control group. Group 4 was more accurate than group 3, but had a significant discrepancy in the cervical third. The first factor that may have affected the results is the matrix design of group 3, which did not have any vertical stops in contrast to those of groups 1 and 2. The landmarks used for the precise fitting were the palatal surface of the tooth and the buccal gingival margin. Palatal and buccal finger pressure were required to achieve the desired fitting; however, this procedure is not standardized and may lead to errors because of the lack of vertical stops and the material’s resistance to flow. These restorations are technique sensitive, and this is the reason why the authors created group 4, where the matrix was seated onto the model by means of the transparent prefabricated plastic tray. This modification has not been previously reported in the literature, and ensures vertical stops and more precise fitting of the matrix. Furthermore, by using the clear tray, pressure was applied in a different direction, which is reflected in the results. The discrepancies in group 4 in the incisal and middle third were not significant in contrast to the cervical bulkier third compared to the control group. The fourth group was more accurate than the third group overall. Moreover, the inaccurate cervical third in group 4 is the easier to correct by the clinician, and has a smaller influence on the esthetic outcome compared to the incisal and middle third. However, the cervical third is crucial to alter in a biologically friendly way because over-contoured restorations impede the maintenance of periodontal health [28].

For groups 1 and 2, the restorative material was injected after the seating of the matrix. Verifying the precise fitting was an easy procedure due to the absence of the restorative material inside and the bilateral extension of the matrix onto the occlusal surfaces of the two premolars. In contrast, for groups 3 and 4, the matrix was seated with the restorative material inside. Therefore, whether the matrix was loaded with composite when the fitting took place is another parameter which may have affected the results.

In the current study, two restorative materials were used: one flowable resin composite for groups 1 and 2 and one conventional preheated resin composite for groups 3 and 4. The viscosity of flowable resin composites permits the filling of the narrow space between the matrix and the tooth surface by exerting minimal stresses onto the matrix. Preheated resin composites at 55 °C increase flowability and adaptability when operation forces are applied, but they keep their shape when operation forces do not exist. However, a quick reduction in temperature was observed after the composite’s removal from the heating device, and this may increase viscosity and affect clinical behavior [29,30,31].

Moreover, the tested materials have different volume polymerization shrinkage rates. Flowable resin composites have higher polymerization shrinkage compared to conventional resin composites. For the flowable composite that was used in this study, the manufacturer provided a volume polymerization shrinkage rate of 3.4%, while, for the conventional composite, that rate was estimated at around 2.09% [32,33]. This may be the reason for some negative values observed for group 2.

Careful and detailed esthetic analysis and design of each case is a major factor in order to achieve the desired outcome. Before the final restoration, a mock-up process must be applied. This tool is the clinical simulation of the final result. By using a mock-up, the clinician verifies esthetics, phonetics and function. The shapes of the teeth, their length, the relationship between the incisal edges and the lower lip, the midline and the color are examined, and, at that stage, the patient can see and accept the final result [27]. In a previous publication, significant differences between the mock-up and the diagnostic wax-up were observed using several popular materials for the index and the mock-up [27]. Since the injection of flowable composite resin in a silicone transparent mold proved to be a quite accurate replication of the wax-up in the present study, it could be proposed to be used for the mock-up procedure as well, to avoid the discrepancies between the mock-up and wax-up previously described [27].

Concerning the limitations of the current study, the range of the values in the control group was not zero, as it would be ideal. The range was expected to be determined from the printer’s nominal precision of 62 μm. However, the initial casts of the researched groups were printed from the same printer, and the interclass correlation coefficient demonstrated acceptable discrepancies among the casts.

Another limitation of this study is the fact that only the volume of the labial surface was evaluated, while the distal and mesial surfaces are very important too. Nevertheless, the researched techniques did not include those surfaces, and their shaping mainly depends on the clinician’s free-hand operation. Group 2 is an exception because the first matrix from which the three first veneers resulted included the proximal surfaces.

Regarding the measurement accuracy of this study, the manufacturer of the 3Shape monitoring software provides uncertainty regarding 50 μm. However, in a published study by Michou et al., the measurement uncertainty was calculated to be ±0.01 mm at a level of confidence of 95%. In this study, the 3D models were dental semi-arches and it was noted that the measurement errors and uncertainty were expected to be smaller for single-tooth 3D models and larger for full-arch 3D models, as in the current study [34]. However, in the current study, a laboratory scanner was used and the STL files were imported into the software for patient monitoring. This fact resulted in truer measurements because of the laboratory scanner’s higher accuracy compared to the intraoral scanner. Both studies used the same method for 3D model alignment, which was the reference surface alignment. The method was proved to provide less measurement errors than the other available alignment methods, such as best fit alignment. Best fit alignment between two objects uses an iterative closest point algorithm, and the objects are brought into a roughly aligned (matched) position. While reference surface alignment provides the restriction of the alignment process to identified surfaces, which, in this research project, were selected to be the surfaces that have undergone no intentional changes. Finally, it is suggested that the measurement uncertainty in the current study was similar to the study by Michou et al. [34], due to their common methodology, and that this level of uncertainty is acceptable for the purpose of this study.

## 5. Conclusions

1. The use of one or two clear silicone matrices and the injection of flowable resin composite (groups 1 and 2) resulted in direct veneers without statistically significant discrepancy compared to the diagnostic wax-up.
2. The technique with a clear matrix for each tooth combined with pre-heated resin composite (group 3) was the most inaccurate group at the incisal and middle third.
3. The use of the clear tray in combination with the matrix for each tooth and the pre-heated resin composite (group 4) improved the accuracy of the technique described in group 3.

## Data Availability

Data Availability Upon Request.
